# Radiation-Induced Alterations in Proliferation, Migration, and Adhesion in Lens Epithelial Cells and Implications for Cataract Development

**DOI:** 10.3390/bioengineering9010029

**Published:** 2022-01-12

**Authors:** Graysen Vigneux, Jake Pirkkanen, Taylor Laframboise, Hallie Prescott, Sujeenthar Tharmalingam, Christopher Thome

**Affiliations:** 1 Biomolecular Sciences Program, Laurentian University, 935 Ramsey Lake Road, Sudbury, ON P3E 2C6, Canada; gvigneux@laurentian.ca (G.V.); sutharmalingam@nosm.ca (S.T.); 2 Department of Biology, Laurentian University, 935 Ramsey Lake Road, Sudbury, ON P3E 2C6, Canada; jpirkkanen@laurentian.ca (J.P.); tlaframboise@nosm.ca (T.L.); hprescott@nosm.ca (H.P.); 3 Northern Ontario School of Medicine, Laurentian University, 935 Ramsey Lake Road, Sudbury, ON P3E 2C6, Canada; 4 Nuclear Innovation Institute, 620 Tomlinson Drive, Port Elgin, ON N0H 2C0, Canada

**Keywords:** ionizing radiation, lens epithelial cell, cataract, proliferation, migration, adhesion

## Abstract

The lens of the eye is one of the most radiosensitive tissues. Although the exact mechanism of radiation-induced cataract development remains unknown, altered proliferation, migration, and adhesion have been proposed as factors. Lens epithelial cells were exposed to X-rays (0.1–2 Gy) and radiation effects were examined after 12 h and 7 day. Proliferation was quantified using an MTT assay, migration was measured using a Boyden chamber and wound-healing assay, and adhesion was assessed on three extracellular matrices. Transcriptional changes were also examined using RT-qPCR for a panel of genes related to these processes. In general, a nonlinear radiation response was observed, with the greatest effects occurring at a dose of 0.25 Gy. At this dose, a reduction in proliferation occurred 12 h post irradiation (82.06 ± 2.66%), followed by an increase at 7 day (116.16 ± 3.64%). Cell migration was increased at 0.25 Gy, with rates 121.66 ± 6.49% and 232.78 ± 22.22% greater than controls at 12 h and 7 day respectively. Cell adhesion was consistently reduced above doses of 0.25 Gy. Transcriptional alterations were identified at these same doses in multiple genes related to proliferation, migration, and adhesion. Overall, this research began to elucidate the functional changes that occur in lens cells following radiation exposure, thereby providing a better mechanistic understanding of radiation-induced cataract development.

## 1. Introduction

The lens of the eye has been said to be among the most radiosensitive tissues in the human body. The International Commission on Radiological Protection (ICRP) provides recommendations on occupational and public dose limits of ionizing radiation to various biological tissues including the lens of the eye. Historically, it was believed that an accumulated lifetime equivalent dose of 15 Sv would cause no vision-impairing opacities, and dose limits to the lens of the eye were set at 300 mSv per year [1]. The recommended annual occupational dose was then lowered to 150 mSv following the release of ICRP Publication 41, based on a new threshold dose estimate of 5 Sv for vision-impairing cataracts [2]. The most recent recommendations, published in 2012 in ICRP Publication 118, suggest that the threshold for cataract formation is even lower at an absorbed dose of 0.5 Gy from low linear energy transfer (LET) radiation [3]. Consequently, the recommended equivalent dose limit to the eye was again lowered to 20 mSv per year, averaged over 5 years, with no single year exceeding 50 mSv [3]. These reductions in thresholds for radiation-induced cataracts have predominantly been the result of recent epidemiological studies.

Radiation-induced cataracts have been studied in various epidemiology cohorts, within which the majority of data come from atomic bomb survivors, radiotherapy patients, and medical workers [4]. A threshold dose for lens opacities of greater than 1 Gy was initially established in atomic bomb survivors, but this threshold has since been lowered to approximately 0.5 Gy when latency times were lengthened to 50 years or more [4]. Data from radiotherapy patients and medical workers yielded similar cataract risk estimates; however, no threshold dose was calculated in these studies [4]. Along with these three cohorts, cataract risk estimates have also been studied in Chernobyl liquidators, diagnostic imaging patients, nuclear workers, astronauts, airline pilots, and residents of contaminated buildings [4]. Despite the abundance of epidemiological studies on radiation-induced cataracts, the exact mechanism by which radiation exposure can lead to lens opacifications still remains unknown.

The lens of the eye is comprised of three main structures: the lens capsule, the lens fiber cells, and the lens epithelium [5]. The elastic lens capsule surrounds the entire lens, is transparent, and is composed of collagen, predominantly Type IV. The bulk of the lens is made up of lens fiber cells, which are tightly packed and transparent. Lastly, the lens epithelium is a simple cuboidal epithelium [5]. This single layer of epithelial cells is located in the anterior portion of the lens between the capsule and fibers. New lens fibers are derived from the equatorial cells of the lens epithelium [5]. The first step in this process is the elongation of the lens epithelial cells (LECs). Once elongated, the cells dissociate from the lens capsule and migrate inwards. During this maturation process, lens fibers lose organelles and their nuclei [5]. The final location of the lens fibers is dictated by their age. Newer lens fibers are located at the outer cortex, while older and more mature lens fibers are located more centrally.

Cataracts are defined as clouding of the lens of the eye which can lead to impaired vision and even vision loss [6]. While cataracts are highly correlated with ageing, other factors can increase the risk of cataract development, such as ocular trauma and metabolic disorders, as well as exposure to ionizing radiation [6]. There are three major types of cataracts, which are defined based on the region in which they are formed: nuclear, cortical, and posterior subcapsular (PSC). Nuclear cataracts develop in the central region of the lens of the eye and are the most common type associated with aging. Cortical cataracts form in the cortex of the lens, which is located peripherally to the nucleus. Lastly, PSC cataracts form in the back of the lens below the capsule. PSC cataracts are known to be the most common type associated with ionizing radiation [6].

Although no concrete mechanism has been established to explain the formation of radiation-induced cataracts, several studies have suggested potential pathways [7,8,9,10,11,12]. These include abnormal differentiation and migration of LECs, where radiation-damaged cells migrate to the posterior region of the lens, resulting in opacities. Ionizing radiation has also been suggested to stimulate the proliferation of the actively dividing LECs. Markiewicz et al. [7] found that proliferation rates were increased in LECs in mice following X-ray exposure of 100 or 250 mGy. These phenotypic changes could result from altered gene and protein expression due to oxidative stress and DNA damage resulting from ionizing radiation. For example, radiation exposure has been shown to alter the expression of cell growth genes including transforming growth factor beta (*TGFβ*) [8], fibroblast growth factor 2 (*FGF2*) [9], matrix metalloproteases (*MMP*) [10], and *CDKN1A* [11].

Although some knowledge has been gained on the effects of ionizing radiation on the lens of the eye and its role in cataractogenesis, there are several areas that still require further investigation. First, most studies have focused on high doses of radiation, so a better comprehension of the effects of lower doses (≤500 mGy) must be established. Secondly, while recent studies have begun to directly investigate the interactions of radiation with the actively dividing LECs, a precise mechanism remains unknown. Lastly, questions remain regarding the timing of phenotypic changes post exposure. Here, we investigated these topics by studying the effects of ionizing radiation on proliferation, migration, and adhesion in cultured LECs.

## 2. Materials and Methods

### 2.1. Cell Culture

Human lens epithelial HLE-B3 cells were purchased from American Type Culture Collection (CRL-11421, ATCC, Manassas, VA, USA). Cells were cultured in Eagle’s minimal essential medium with Earle’s salt (MT10010CV, Fisher Scientific, Hampton, NH, USA), supplemented with 20% fetal bovine serum (SH3039603, Fisher Scientific), 1% penicillin–streptomycin (15-070-063, Fisher Scientific), and 1 mM sodium pyruvate (11-360-070, Fisher Scientific). Cells were maintained in a humidified incubator at 37 °C and 5% CO_2_.

### 2.2. Irradiation

Cells were irradiated with X-rays using an X-Rad 320 irradiation cabinet (Precision X-ray, Madison, CT, USA) operated at 320 kV and 5.6 mA with a 2 mm aluminum filter. Cells were irradiated on cold phosphate-buffered saline (PBS). Cells were exposed to a dose of 0.1, 0.25, 0.5, 1, or 2 Gy at a mean dose rate of 0.68 Gy/min. Absorbed doses were verified using LiF thermoluminescent dosimeters (Mirion Technologies, Atlanta, GA, USA). Control cells were sham-irradiated and handled in parallel with the treatment flasks. Following irradiation, cells were incubated for either 12 h or 7 day before analysis.

### 2.3. Proliferation

The 3-(4,5-dimethylthiazol-2-yl)-2,5-diphenyltetrazolium bromide (MTT) assay was performed to assess the proliferative ability of irradiated and unirradiated cells. This assay quantifies the colorimetric change resulting from the reduction of MTT by metabolically active cells. Although this assay does not directly measure proliferation, it is commonly used as a surrogate for measuring changes in cell growth [13,14]. For the 12 h timepoint, cells were irradiated directly in 96-well plates (07-201-94, Fisher Scientific). In each well, a 100 µL cell suspension with a density of 7.5 × 10^4^ cells/mL was seeded and incubated for 2 days prior to irradiation. For the 7 day timepoint, 1.5 × 10^5^ cells were initially seeded in a T25 flask 24 h prior to irradiation. On day 5 of the 7 day post-treatment incubation period, cells were dissociated from their T25 flasks. Each well of a 96-well plate was seeded with a 100 µL cell suspension with a density of 7.5 × 10^4^ cells/mL and incubated for 2 days.

Following both incubation periods (12 h or 7 day), 10 µL of MTT (M2128, Sigma-Aldrich, Oakville, ON, CA) at a concentration of 5 mg/mL was added to each well and incubated at 37 °C for 3.5 h. Following the incubation period, the culture medium was replaced with 100 µL of dimethyl sulfoxide (85190, Sigma-Aldrich) and incubated in a dark room for 15 min. The optical density (OD) value of each well was then quantified using a Synergy HTX multi-mode microplate reader (BioTek, Winooski, VT, USA) at a wavelength of 570 nm. The experiments were performed in triplicate for each incubation period. Within each replicate, three wells were seeded for each dose.

### 2.4. Migration

Cell migration was measured using the Boyden chamber transwell migration assay. Cells were seeded into T25 flasks 24 h prior to irradiation. A total of 1.0 × 10^6^ cells were seeded for the 12 h timepoint and 1.5 × 10^5^ cells for the 7 day timepoint. Following the post-irradiation incubation period (12 h or 7 day), cells were trypsinized, centrifuged for 7 min at 335 *g*, and resuspended in serum-free media. A 1 mL cell suspension with a density of 1 × 10^5^ cells/mL was seeded in the upper chamber of the transwell insert (08-771-21, ThermoFisher Scientific, Waltham, MA, USA) while 600 µL of complete media was added to each well of a 24-well plate below the insert. Following an 8 h migration period, cells on the upper membrane of the insert were removed using a cotton swab. The cells that had migrated to the bottom of the insert were then washed twice with PBS and fixed for 20 min at room temperature with ice-cold methanol (A452-4, Fisher Scientific, Waltham, MA, USA). Following fixing, inserts were washed once again with PBS and stained with 0.3% crystal violet (C0775, Sigma-Aldrich) for 20 min at room temperature. Cells were then imaged using an EVOS XL Core Imaging System (AMEX1000, Fisher Scientific) and counted using the image processing program ImageJ version 1.52 a [15]. The experiments were performed in triplicate for each timepoint. Within each replicate, three transwell inserts were seeded for each dose.

Cell migration was also measured using the wound-healing assay. For the 12 h timepoint, cells were irradiated directly in 35 mm Petri dishes (07-202-514, Fisher Scientific). In each plate, a 3 mL cell suspension containing 6.0 × 10^5^ cells was seeded and incubated for 24 h prior to irradiation. For the 7 day timepoint, 2.0 × 10^5^ cells were initially seeded in a T25 flask 24 h prior to irradiation. On day 6 of the 7 day post-treatment incubation period, cells were dissociated from their T25 flasks and a 3 mL cell suspension containing 6.0 × 10^5^ cells was seeded into a 25 mm Petri dish for the final 24 h.

Following both incubation periods (12 h or 7 day), a scratch was introduced to each Petri dish using a 10 µL sterile pipette tip. The dishes were washed twice with sterile PBS to remove the cells from the scratched region and were then continually cultured in a complete medium. Images of the scratch were captured using a Cytation 5 digital microscope (BioTek, USA) at 0, 12, 24, 36, and 48 h post-scratch. The average wound width at four different points along the scratch was measured to determine wound closure rate. The experiments were performed in triplicate for each timepoint. Within each replicate, three Petri dishes were seeded for each dose.

### 2.5. Adhesion

Cell adhesion was measured on three different extracellular matrices (ECM): fibronectin (F2006, Sigma-Aldrich), laminin (C5533, Sigma-Aldrich), and human type IV collagen (L2020, Sigma-Aldrich). 24-well plates (09-761-146, Fisher Scientific) were coated with 300 µL of each ECM reconstituted in PBS for 18 h. The coating concentration of type IV collagen was 6 μg/cm^2^ while that of fibronectin and laminin was 1 μg/cm^2^.

Initially, cells were seeded into T25 flasks 24 h prior to irradiation. A total of 4.0 × 10^5^ cells were seeded for the 12 h timepoint and 1.0 × 10^5^ cells for the 7 day timepoint. Following the post-irradiation incubation period (12 h or 7 day), cells we trypsinized and a 300 µL cell suspension containing 1 × 10^5^ cells was seeded on each ECM. Cells were allowed to adhere to the ECM for 120 min at 37 °C. Following the incubation period, the wells were washed with PBS to remove unadhered cells. Adhered cells were then dissociated with trypsin–EDTA and counted using a hemocytometer. The experiments were performed in triplicate for each incubation period and within each replicate, three wells were seeded for each dose.

### 2.6. Gene Expression

Total RNA extraction, cDNA synthesis, and design and validation of RT-qPCR primers were performed as previously described [16]. RT-qPCR experiments were performed using the Quantstudio 5 qPCR instrument (ThermoFisher Scientific, Waltham, MA, USA). The primer sequences for each of the genes of interest are shown in Table 1. The final 15 µL reaction mix was composed of 2× Luna^®^ Universal qPCR mastermix (10096444, New England BioLabs, Ipswich, MA, USA), 600 nM forward and reverse primers, and 6 ng of input cDNA. The following qPCR protocol was followed for 40 cycles: 95 °C for 15 s, and 60 °C for 30 s, and then readout of the plate data. Amplicon melt-curve analysis was run to validate single amplicon specificity following these 40 cycles. Cycle threshold (C_T_) data were analyzed using the QuantStudioTM Design and Analysis Software v1.4.1 (Applied Biosystems, Waltham, MA, USA). Samples were normalized to the mean of three control housekeeping genes: RSP13, RPS18, and RPL4. Relative expression of genes was calculated utilizing the ΔΔC_T_ method [17] using the following formula:

(1) $$2^{\Delta \Delta C_{T}}=2^{\left(\Delta_{C_{T}}gene-\Delta_{C_{T}}housekeeping\right)}$$

### 2.7. Statistical Analysis

Statistical analyses were performed with GraphPad Prism version 8.4.3 (San Diego, CA, USA). All endpoints were run in three independent experimental replicates. Data were compared across radiation doses using a one-way ANOVA followed by Dunnett’s multiple comparisons test, with *p* < 0.05 considered statistically significant.

## 3. Results

### 3.1. Proliferation

**Figure 1:**
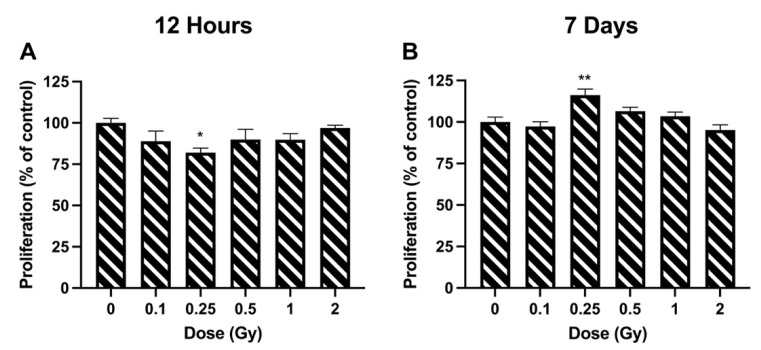
Cell proliferation following ionizing radiation exposure in HLE-B3 cells. Proliferation was quantified using the MTT assay at 12 h (**A**) and 7 days (**B**) post exposure. Data are presented as a percentage of unirradiated control cell proliferation. Bars represent the average of three independent experimental replicates ± SEM. Data were analyzed using a one-way ANOVA followed by Dunnett’s multiple comparisons test (* *p* < 0.05, ** *p* < 0.01).

The effects of radiation on cell proliferation were observed by analyzing metabolic activity via the MTT assay. A significant decrease in cell proliferation was observed at a dose of 0.25 Gy, where proliferation was 82.06 ± 2.66% of unirradiated levels (Figure 1A). No significant difference was identified at any of the other doses. Similar to the 12 h timepoint, a dose of 0.25 Gy was the only exposure level that produced a significant change in proliferation at 7 days. However, in this case, proliferation rates were increased to 116.16 ± 3.64% of controls (Figure 1B).

### 3.2. Migration

**Figure 2:**
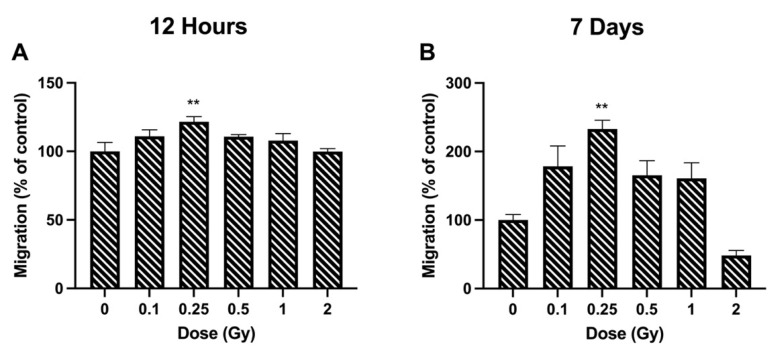
Cell migration following ionizing radiation exposure in HLE-B3 cells. Migration was quantified using the Boyden chamber assay at 12 h (**A**) and 7 days (**B**) post exposure. Data are presented as a percentage of unirradiated control cell migration. Bars represent the average of three independent experimental replicates ± SEM. Data were analyzed using a one-way ANOVA followed by Dunnett’s multiple comparisons test (** *p* < 0.01).

The effects of radiation on cell migration were studied using the transwell migration assay. Cell migration was significantly increased at a radiation dose of 0.25 Gy, where rates were 121.66 ± 6.49% of control cells (Figure 2A). No significant change was observed at the other doses. At 7 days post irradiation, again a dose of 0.25 Gy significantly increased migration. The increase was much greater at 7 days compared to 12 h, at 232.78 ± 22.22% of control cells (Figure 2B). There was a trend towards increased migration at 0.1, 0.5, and 1 Gy, and a decrease at 2 Gy, but this was not significant.

**Figure 3:**
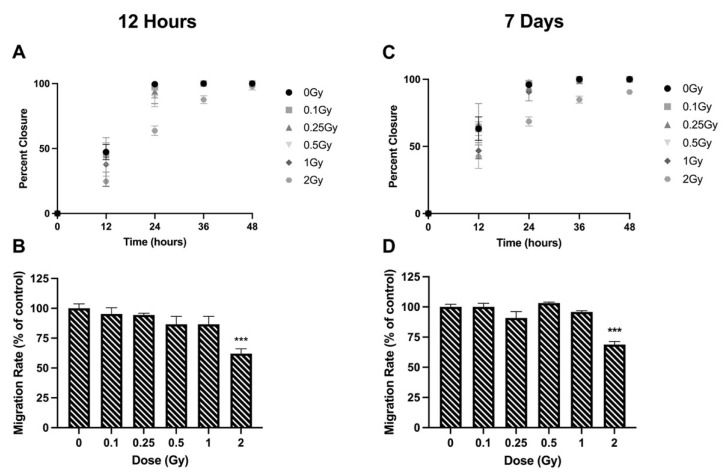
Cell migration following ionizing radiation exposure in HLE-B3 cells. Migration was quantified using the wound-healing assay at 12 h (**A**,**B**) and 7 days (**C**,**D**) post exposure. The percent of wound closure was measured every 12 h up to 48 h post scratch (**A**,**C**). The average rate of wound closure across the first 24 h was calculated and was plotted as a percentage of unirradiated controls (**B**,**D**). Data points represent the average of three independent experimental replicates ± SEM. Data were analyzed using a one-way ANOVA followed by Dunnett’s multiple comparisons test (*** *p* < 0.001). Note: data points overlap one another at 36 and 48 h once wounds reach full closure (**A**,**C**).

Migration of lens epithelial cells was also measured using the wound-healing assay. Migration rates were tracked for 48 h. When looking at the migration rate 12 h post irradiation, control cells migrated at a rate of 13.16 ± 0.49 µm/h (Figure 3A). Radiation doses of 0.1–1 Gy did not impact the rate of migration (Figure 3A,B). However, a dose of 2 Gy resulted in a significant migration inhibition, with rates that were 62.05 ± 4.09% of controls. The same trend in migration rate was observed 7 days post irradiation. The speed of migrated control cells was 11.50 ± 0.26 µm/h (Figure 3C). No significant differences were observed at 0.1–1 Gy, but 2 Gy reduced migration rates to 68.75 ± 2.60% of controls (Figure 3C,D).

### 3.3. Adhesion

**Figure 4:**
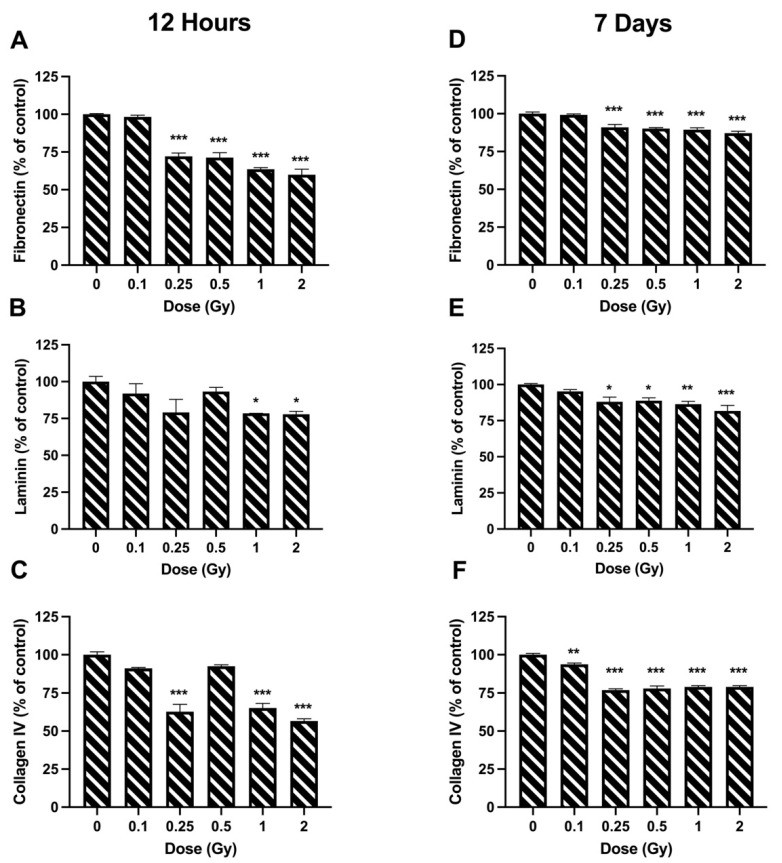
Cell adhesion following ionizing radiation exposure in HLE-B3 cells. Adhesion was quantified on three different extracellular matrices (fibronectin, laminin and collagen IV) at 12 h (**A**–**C**) and 7 days (**D**–**F**) post exposure. Data are presented as a percentage of unirradiated control cell adhesion. Bars represent the average of three independent experimental replicates ± SEM. Data were analyzed using a one-way ANOVA followed by Dunnett’s multiple comparisons test (* *p* < 0.05, ** *p* < 0.01, *** *p* < 0.001).

The effects of radiation on cell adhesion were studied by analyzing three different extracellular matrices. In general, radiation tended to reduce adhesion rates on all three matrices. When looking at 12 h post irradiation, a significant drop was observed at all doses greater than 0.25 Gy on fibronectin, where adhesion was between 59–72.14% of controls (Figure 4A). There was also a significant decrease in adhesion following 1 and 2 Gy on laminin, where adhesion was 78.46 ± 0.08% and 77.46 ± 1.98% of controls respectively (Figure 4B). Furthermore, a significant decrease was observed following 0.25, 1, and 2 Gy on human type IV collagen, where adhesion was between 56.58–65.20% of controls (Figure 4C). A similar trend was observed at 7 days post irradiation; however, the magnitude of the decrease in adhesion tended to be larger at 12 h. Again, all doses over 0.25 Gy caused a reduction in adhesion to fibronectin, where the rates were between 87.12–90.26% of controls (Figure 4D). The same occurred for laminin, where adhesion was between 81.69–88.85% of controls. On type IV collagen, all doses significantly decreased adhesion, where rates were between 76.88–93.68% of controls.

### 3.4. Gene Expression

**Figure 5:**
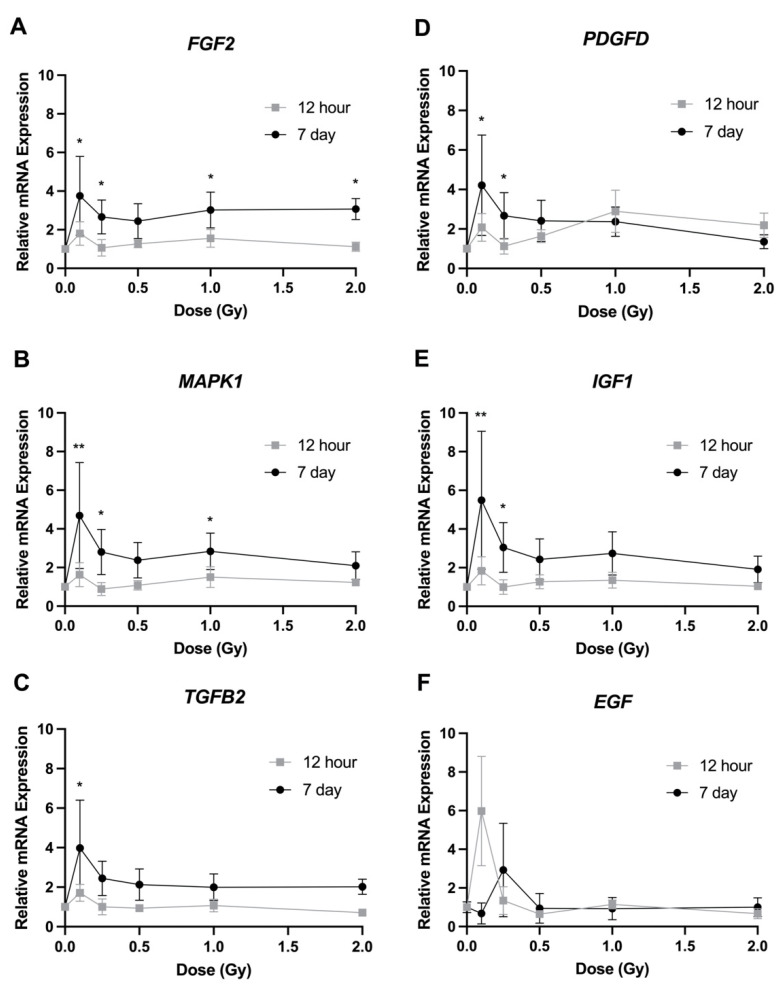
Relative transcript expression of genes related to cell proliferation in HLE-B3 cells following ionizing radiation exposure. Grey squares represent 12 h post irradiation data, while 7 day post irradiation data are represented by black circles. Data are presented as a relative expression compared to unirradiated control cells. Data points represent the average of three independent experimental replicates ± SEM. Data were analyzed using a one-way ANOVA followed by Dunnett’s multiple comparisons test (* *p* < 0.05, ** *p* < 0.01). Abbreviations: (**A**) Fibroblast Growth Factor 2 (FGF2); (**B**) Platelet Derived Growth Factor D (PDGFD); (**C**) Mitogen-Activated Protein Kinase 1 (MAPK1); (**D**) Insulin-Like Growth Factor 1 (IGF1); (**E**) Transforming Growth Factor Beta 2 (TGFB2); (**F**) Epidermal Growth Factor (EGF).

The effects of radiation on gene expression were studied using RT-qPCR. Three different gene panels were studied to identify expression changes in genes related to cell proliferation, migration, and adhesion. When looking at the expression levels of the proliferation gene panel (*FGF2*, *MAPK1*, *TGFB2*, *PDGFD*, *IGF1*, *EGF*), no significant changes were observed across all the radiation doses at the 12 h timepoint. However, consistent expression changes were identified at the 7 day timepoint, particularly at the lowest doses of 0.1 and 0.25 Gy. *FGF2* levels were significantly increased at 0.1, 0.25, 1, and 2 Gy by 2.4–3.8 fold compared to controls (Figure 5A). Similarly, *MAPK1* showed increased expression at 0.1, 0.25, and 1 Gy with a range of 2.8–4.7 fold (Figure 5B). *TGFB2*, *PDGFD*, and *IGF1* all showed significantly increased expression at 0.1 and 0.25 Gy only (Figure 5C–E). Finally, no significant changes were identified in *EGF* (Figure 5F).

**Figure 6:**
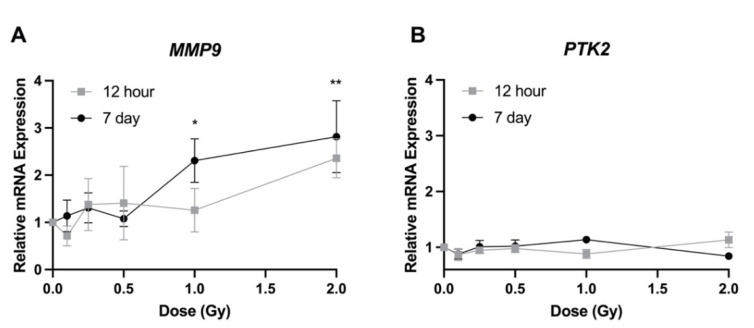
Relative transcript expression of genes related to cell migration in HLE-B3 cells following ionizing radiation exposure. Grey squares represent 12 h post irradiation data, while 7 day post irradiation data are represented by black circles. Data are presented as a relative expression compared to unirradiated control cells. Data points represent the average of three independent experimental replicates ± SE. Data were analyzed using a one-way ANOVA followed by Dunnett’s multiple comparisons test (* *p* < 0.05, ** *p* < 0.01). Abbreviations: (**A**) Matrix Metallopeptidase 9 (MMP9); and (**B**) Protein Tyrosine Kinase 2 (PTK2).

A different trend was observed when looking at genes related to cell migration. At 7 days post irradiation, expression of *MMP9* was significantly increased following 1 and 2 Gy by 2.3- and 2.8-fold respectively, but no changes were seen at lower doses (Figure 6A). *MMP9* expression was unchanged at the 12 h timepoint. When looking at the gene expression of *PTK2*, no significant change in expression was observed at 12 h or 7 days post irradiation (Figure 6B).

**Figure 7:**
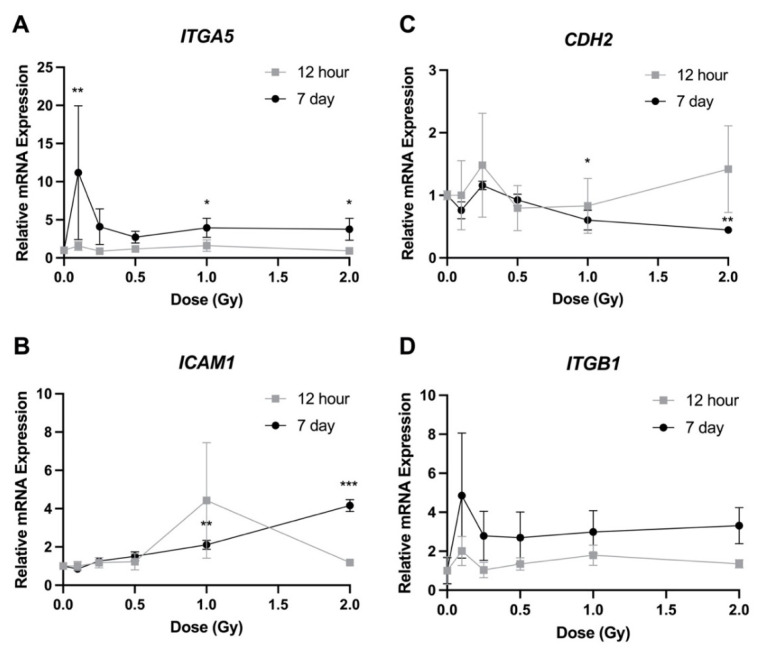
Relative transcript expression of genes related to cell adhesion in HLE-B3 cells following ionizing radiation exposure. Grey squares represent 12 h post irradiation data, while 7 day post irradiation data are represented by black circles. Data are presented as a relative expression compared to unirradiated control cells. Data points represent the average of three independent experimental replicates ± SE. Data were analyzed using a one-way ANOVA followed by Dunnett’s multiple comparisons test (* *p* < 0.05, ** *p* < 0.01, *** *p* < 0.001). Abbreviations: (**A**) Integrin Subunit Alpha 5 (*ITGA5*); (**B**) Intercellular Adhesion Molecule 1 (*ICAM1*); (**C**) Cadherin 2 (*CDH2*); (**D**) Integrin Subunit Beta 1 (*ITGB1*).

Expression of genes related to adhesion varied considerably depending on the gene. No expression changes were observed 12 h post irradiation for any of the four genes (Figure 7). At 7 days post exposure, *ITGA5* showed a large increase in expression of 11.2-fold following 0.1 Gy (Figure 7A). A more modest increase was also observed at 1 and 2 Gy. Both *ICAM1* and *CDH2* also had a significant increase in expression following 1 and 2 Gy exposures, with *ICAM1* increasing by 2.1- and 4.2-fold and *CDH2* increasing by 2.1- and 4.2-fold respectively (Figure 7B,C). No significant difference in gene expression was observed at any dose when looking at *ITGB1* (Figure 7D).

In summary, a consistent pattern was identified in genes related to proliferation. Expression was increased at low doses before returning to baseline levels at high doses. Expression patterns of genes related to migration and adhesion were more variable. Some showed a more classical dose response pattern, with gene expression increasing with dose (*MMP9*, *ICAM1*), whereas others showed increases only at low doses (ITGA5), and finally some showed no significant changes. Across all three gene panels, expression changes were more pronounced at the 7 day timepoint compared to 12 h.

## 4. Discussion

The goal of this study was to identify radiation-induced functional changes in LECs 12 h and 7 days post exposure to elucidate the initiating mechanisms of cataractogenesis. HLE-B3 cells were exposed to an acute X-ray dose between 0.1–2 Gy. Assays were conducted to observe changes in proliferation, migration, and adhesion, and qPCR was performed to identify transcriptional changes related to each process. Interestingly, our results showed that a dose of 0.25 Gy had a significant impact on cell function consistently across all experimental assays. With respect to proliferation and migration, these stimulatory effects were not observed at the higher doses of 1 and 2 Gy. Adhesion, on the other hand, was consistently reduced at doses ≥0.25 Gy. Most of the effects were more pronounced at the 7 day timepoint compared to the 12 h one. These cellular changes can at least in part be explained by the transcriptional alterations that were observed.

We observed a contrasting response to radiation with cell proliferation at the 12 h vs. 7 day timepoints. A dose of 0.25 Gy resulted in a decrease in proliferation 12 h post irradiation, which was followed up by an increase in proliferation 7 days post irradiation. The effects of ionizing radiation on LEC proliferation have been previously studied by other groups [7,12,18]. Similar to our experiments, Fujimichi and Hamada [12] looked at LECs in culture, but they calculated proliferation based on the relative size of clonogenic colonies. They identified increased proliferation, but only in doses ≥2 Gy. On the other hand, Markiewicz et al. [7] examined cell density in the lenses of mice exposed to X-rays in vivo. Their results correlate closely with our 7 day post irradiation proliferation results (Figure 1B), which indicated that proliferation is stimulated by a dose of 0.25 Gy, with rates dropping back down to baseline levels at higher doses. However, these in vivo results were identified after only 24 h post irradiation. Furthermore, it was shown in the lens of rabbits that following high-dose ionizing radiation exposure, mitotic activity ceases within a few hours and up to 4 days post irradiation [18]. Following this period of inactivity, cells displayed an increase in cellular division for a period of 7 days before they then returned to pre-exposure levels [18]. This trend matches what we identified, albeit at a lower dose of 0.25 Gy, where LEC proliferation significantly dropped 12 h post irradiation and proceeded to increase 7 days post exposure.

The effects of transcriptional changes on the proliferation rate of LECs have been reported across a variety of publications [9,19,20,21,22,23,24,25,26,27,28]. Several of these have looked at the effects of growth factors such as FGF2, IGF, EGF, and PDGFD. In unirradiated LECs, all of these genes can lead to an increased level of proliferation when overexpressed [22,23,24,25,27]. Our data identified an increase in the expression of *FGF2*, *PDGFD*, and *IGF1*, but not *EGF*, at 0.25 Gy 7 days post irradiation (Figure 5), which matched our proliferation data (Figure 1B). This suggests that low-dose radiation can lead to overexpression of certain growth factors, resulting in stimulated proliferation. This correlates with the finding of Chang et al. [6], who they observed an increase in *FGF2* expression post irradiation when HLE cells underwent 4 Gy irradiation. Our data also showed a significant increase in *MAPK1* expression 7 days post irradiation, suggesting a role for MAPK1 in this response. The deletion of MAPK1 has been shown to decrease proliferation in LECs [26,28]. In other cell types, low-dose radiation has been shown to increase expression of these same genes, resulting in stimulated proliferation [29,30,31]. We did identify increased expression levels at doses other than 0.25 Gy, mainly at 0.1 Gy (Figure 5); however, we did not observe an increase in proliferation at these doses. We also did not observe any significant transcriptional changes at 12 h post exposure. This suggests that additional pathways must be involved in these responses.

Alteration in LEC migration has been proposed as a potential mechanism for the formation of PSC cataracts [6]. During the normal process of differentiation, LECs in the bow region will migrate medially towards the lens nucleus. However, in rats exposed to approximately 20 Gy of gamma radiation, LECs were found to follow a different migration path and moved towards the posterior region of the lens [32]. The overall rate of migration in the rat lenses was also reduced post irradiation. This differs from what was shown in the present study, where radiation stimulated cell migration (Figure 2). However, the radiation doses were different by several orders of magnitude (0.25 vs. 20 Gy) and migration effects were studied over different timescales (12 h and 7 day vs. 12 week). To the best of our knowledge, no previous studies have documented cell migration changes in LECs in the low-dose region.

Various genes are involved in the migration of LECs [19,21,33,34,35]. We observed an increased expression of *MMP9* at 1 and 2 Gy (Figure 6A). An increase in MMP9 protein levels has previously been identified following proton irradiation [10]. Liu et al. [35] showed that an increase in TGFβ2 expression significantly increased migration and that inhibition of PTK2 decreased TGFβ2-mediated cell migration, indicating that both genes have a role in the migration of LECs. In the present study, no significant changes were observed in the expression of *PTK2* (Figure 6B), suggesting that this gene does not play a role in the changes in migration following low doses of radiation. On the other hand, *TGFB2* expression was significantly increased at 0.25 Gy 7 days post irradiation (Figure 5C). Certain growth factors, in particular FGF2 and EGF, are known to play a role in the migration of LECs [28,34]. No significant changes in *EGF* expression were observed; however, FGF2 was significantly increased at 0.25 Gy 7 days post irradiation (Figure 5A). As previously mentioned, *FGF2* expression levels have been shown to increase post irradiation [9].

Ionizing radiation has been shown to alter the adhesion of epithelial cells. Park et al. [36] investigated the effects of 2 Gy ionizing radiation on human mammary epithelial cells and concluded that ionizing radiation causes disruption in extracellular matrix interactions. In our study, we established that 12 h post irradiation, higher doses of ionizing radiation (≥1 Gy) decreased cells’ ability to adhere to all three extracellular matrices (Figure 3A–C). Furthermore, 7 days post irradiation, these effects were seen at doses as low as 0.25 Gy (Figure 3D–F).

Several publications have investigated the effects of gene expression on the adhesion of LECs [37,38,39,40,41]. A reduction in cell attachment to both laminin and collagen was observed when anti-β1 integrin monoclonal antibody (mAb) and anti-ICAM1 mAb were added to the cell growth medium [37]. This would indicate that both ITGB1 and ICAM1 are important for LEC adhesion to the lens capsule. Interestingly, our results suggested the opposite effect following radiation exposure, where *ICAM1* expression increased significantly at higher doses 7 days post irradiation (Figure 7B) but cell adhesion was reduced (Figure 4). Furthermore, we saw no significant changes in *ITGB1* expression following radiation treatment (Figure 7D), which would indicate that ITGB1 does not play a role in the loss of adhesion due to ionizing radiation. Matrix metalloproteinases have been seen to play a role in cell adhesion. When HLE-B3 cells were treated with proteasome inhibitors, both MMP2 and MMP9 were downregulated in the cells, which led to a loss in adhesion [41]. We observed an increase in *MMP9* expression post irradiation at 1 and 2 Gy (Figure 6A), but adhesion was reduced at those same doses (Figure 4). This same response was observed for ITGA5, which has previously been shown to be expressed in LECs and involved in the process of cellular adhesion [39,40]. Lastly, studies using conditional knockouts have shown that both E-cadherin and N-cadherin are involved in LEC adhesion [36]. We identified a significant decrease in expression of *CDH2* (N-cadherin) at higher doses 7 days post irradiation, which correlated with our data showing a decrease in adhesion on all three extracellular matrices at these same doses and at this timepoint (Figure 4).

Alterations in cell proliferation, migration, and adhesion have all been proposed as potential mechanisms for radiation-induced cataracts. Indeed, we show here that both low and high doses can alter these processes in cultured LECs. Based on these results, all three of these processes may be involved in the formation of lens opacities following radiation exposure. For adhesion, there appears to be a threshold at 0.25 Gy, with a consistent reduction in adhesion occurring at doses above this. However, for proliferation and migration, a nonlinear trend, was observed with significant effects occurring at 0.25 Gy, but not at doses above or below. There is currently debate over the threshold dose for radiation-induced cataracts. The ICRP has determined a new threshold of 0.5 Gy based on atomic bomb survivor data [4]. It is difficult to infer how changes in cultured LECs might correlate to alterations in the transparency of whole lenses. Nonetheless, the alterations in adhesion we identified are in line with a threshold dose of several hundred mGy. On the other hand, migration and proliferation data suggests that doses between 0.5–2 Gy have minimal impact.

In this study, we observed a nonlinear dose response when looking at proliferation and migration, where radiation effects peaked at 0.25 Gy and dropped back to control levels at higher doses (0.5–2 Gy). This same parabolic trend was also observed in the expression of several genes. The phenomenon whereby biological systems respond differently to low vs. high doses of radiation is not unique to this study. Many groups have shown J-shaped or U-shaped dose–response curves in other model systems when looking at endpoints such as growth [42], carcinogenesis [43,44], or immune function [45]. A full understanding of these nonlinear responses has not yet been established, but potential mechanisms could include upregulation of DNA repair and antioxidant pathways, heat shock responses, selective apoptosis of aberrant cells, suppression of cancer-promoting inflammation, and stimulation of anticancer immunity [46].

There are several limitations of the design on this study. First, experiments were conducted using the HLE-B3 cell line, which is an immortalized cell line. These cells may respond differently to exogenous stressors, such as ionizing radiation, compared to primary LECs. However, the authors elected to choose this model system because an immortalized cell line was more conducive to the wide range of endpoints, doses, and timepoints that we wanted to investigate. Furthermore, the HLE-B3 cell line is known to maintain many of the functional characteristics of primary LECs [47]. Second, radiation-induced cataracts are known to have a latency period of many months to years before development post exposure. However, it is not realistic to conduct in vitro studies where cells are cultured for multiple years post exposure. We elected to study the radiation-induced functional changes in LEC 12 h and 7 days post exposure. The rationale behind this was to examine the initiating effects following irradiation (12 h timepoint), as well as the persistent phenotypic changes after cells had recovered from the initial radiation stress and had gone through several generations (7 day timepoint).

## 5. Conclusions

In conclusion, HLE-B3 cells were used to explore radiation-induced functional changes following low- and high-dose exposures. We demonstrated that 0.25 Gy irradiation resulted in a decrease in proliferation 12 h post irradiation, followed by an increase 7 days post irradiation. At the same dose, migration was increased at both timepoints. Lastly, cell adhesion was consistently reduced above doses of 0.25 Gy at both timepoints and on all three extracellular matrices. Several genes were analyzed to understand the pathways involved in these functional changes. Our results suggest that FGF2, MAPK1, TGFB2, PDGFD, IGF1, MMP9, ITGA5, ICAM1, and CDH2 all likely play a role in the observed changes in proliferation, migration, and adhesion. Overall, this study has further elucidated some of the functional changes implicated in the process of cataractogenesis in response to low doses of ionizing radiation. Further research is required to determine how these radiation-induced cellular alterations may correlate to lens opacifications in vivo to better understand the risk and threshold level for PSC development.

## Figures and Tables

**Table 1:** Forward and reverse primer sequences for the genes analyzed using RT-qPCR. All primer sequences have an annealing temperature of 60 °C.

Gene	Primer Sequence	
	Forward	Reverse
Proliferation		
*FGF2*	GAGCGACCCTCACATCAAGC	ATAGCCAGGTAACGGTTAGCAC
*TGFB2*	GTGCTTTGGATGCGGCCTA	GGCATGCTCCAGCACAGAA
*IGF1*	ACCCGGAGTACTTCAGCGC	CACAGAAGCTTCGTTGAGAA
*EGF*	GAGATGGGTGTCCCAGTGTG	GGGGTGGAGTAGAGTCAAGACA
*PDGFD*	CTCAGGCGAGATGAGAGCAAT	GCACGTAGCCGTTTCCTTTC
*MAPK1*	ATCTTAAATTTGTCAGGACAAGGG	AGACAGGACCAGGGGTCAA
Migration		
*MMP9*	CTTTGAGTCCGGTGGACGAT	TCGCCAGTACTTCCCATCCT
*PTK2*	TGGGCGGAAAGAAATCCTGC	GGCTTGACACCCTCGTTGTA
Adhesion		
*ITGA5*	GTCGGGGGCTTCAACTTAGAC	GCACACTGACCCCGTCTG
*ITGB1*	ACCGTAGCAAAGGAACAGCA	TCTGTGGCTCCCCTGATCTT
*ICAM1*	TTGAGGGCACCTACCTCTGT	GATAGGTTCAGGGAGGCGTG
*CDH2*	ATGGGAAATGGAAACTTGATGGC	CAGTTGCTAAACTTCACTGAAAGG
Housekeeping		
*RSP13*	CTTTCGTTGCCTGATCGCCG	TCAACTTCAACCAAGTGGGGA
*RSP18*	ATTAAGGGTGTGGCCGAAG	GGTGATCACACGTTCCACCT
*RPL4*	CACGCAAGAAGATCCATCGC	CCGGAGCTTGTGATTCCTGG

## Data Availability

The data presented in this study are available on request to the corresponding author.
